# A comprehensive review of advanced technologies and methods for biodiesel production from microalgae integrating wastewater

**DOI:** 10.3389/fmicb.2026.1980036

**Published:** 2026-10-13

**Authors:** Sonali Biswas, Kaajal Anil Rai, Sanatan Ramnath Iyer, I. Ganesh Moorthy

**Affiliations:** 1 School of Bio-Sciences and Technology, Vellore Institute of Technology, Vellore, Tamilnadu, India; 2 School of Chemical Engineering, Vellore Institute of Technology, Vellore, Tamilnadu, India

**Keywords:** biodiesel, biofuels, microalgae, transesterification, wastewater

## Abstract

Microalgae are third generation feedstock and an alternative for the development of biodiesel through sustainable application which are environment friendly. This paper delves in utilization of microalgae for biodiesel and other value-added products. The microalgae cultivation procedure can become cost effective by utilizing wastewater from domestic and industrial origin for the purpose of biomass and subsequent lipid production. This paper addresses all the breakthrough of microalgae in biotechnological applications and also how machine learning and artificial intelligence can be applied to make the process more favourable and technoeconomic analysis of harvesting and other process has also been discussed. The growth of microalgae and different methods and equipment to achieve high amount of biomass is explored efficiently. This paper emphasizes the use of heterogenous catalyst instead of homogenous because of its reusability and the easy availability of precursors and calcium oxide sources in the environmental wastes like oyster shells, egg shells and waste animal bones. Overall, this chapter delves into the advantages and challenges of utilizing microalgae for the process of biodiesel production and provides alternative methods to address and minimize those challenges for sustainable and economically feasible procedures.

## 1 Introduction

Microalgae are acknowledged as a powerful, nature-based solution for combating wastewater pollution problems, while producing important by-products like renewable fuels, more so, without demands like farmland or freshwater habitats. Their ability to recycle nutrients and carbon dioxide into biomass also makes them an ideal input for sustainable systems, especially in regions which are resource deficient. These microorganisms, worldwide, have been utilized in solving environmental issues (Song, Liu, et al., 2024) by harnessing sunlight, converting solar energy, and using carbon dioxide from air or exhausts from industries/factories, and excess nutrients, in turn forming a nutrient-dense biomass (Enamala et al., 2020). In contrast to land crops, they outpace them by growing rapidly by almost 10–100 times faster, and have more adaptability and flexibility to fluctuating conditions (Novoveská et al., 2023), as well as, for example, salty or nutrient-heavy wastes. They therefore yield outputs at much higher margins per unit area and are also essential for strategic bio-circular systems and pathways (Hu et al., 2023). This sets up integrated plants for wastewater treatment and bioremediation, with a bonus of biofuel creation, pushing aside the pitfalls of traditional crop-based energy sources integration (Singh et al., 2022). Brimming with high levels of nitrogen (up to 50 mg/L), phosphorus (5–20 mg/L) and other organics and particles in the wastewater from urban, domestic and industrial sources, are all responsible for toxic algal blooms, killing fish via oxygen deprivation, consequently disrupting the river ecosystems when introduced untreated (Hosny et al., 2025). Standard treatment plants or STPs require very heavy electricity inputs for aeration (around 30–50% of the costs), for flocculants and other chemical additives, and for massive volumes of sludge disposal needs. All of this worsens the pollution cycles, most of the time (Zhang et al., 2022). Phycoremediation which is when algae consume the macronutrients from the wastewater during their growth phase, also leads to BOD levels dropping by 70–90%, COD by 60–85% and stabilising the swinging pH from 6–9 to 7–8, all the while yielding biomass rich in lipids and oils, driving it toward biodiesel conversion. This successfully reduces the costs by 40–60% as compared to the various chemical methods (Wang et al., 2024).

From kingdoms like Chlorophyta or eukaryotic greens, to Bacillariophyta or silica diatoms, and prokaryotic cyanobacteria, microalgae are known to be vital anchors to aquatic food chains because of sunlight fixing and its conversion into energy via photosynthesis (Sivaramakrishnan et al., 2022). The taxonomy depends on DNA markers or 18S rRNA, pigment profiles (chlorophyll a/b in greens and fucoxanthin in diatoms), and motility by flagella count, as well as spore types for storage of energy in the form of starch or oils (Amin et al., 2023). This diversity is not limited to just taxonomic behaviours and characteristics, but also the metabolic nature, which helps them adapt and colonise extremes like hypersaline brine conditions, acidic drains, nutrient floods, which is done by switching between photoautotrophic (sunlight + CO₂) and mixotrophic nature (eating organics as well) (Kaur, 2025). Wastewater strains are usually known to undergo hybridisation for desired traits for efficient and robust pollutant handling. With their absorption and assimilation abilities of nutrients like nitrogen and phosphorus, which contribute highly to water pollution from domestic and industrial effluents, microalgae dominate the field (Plöhn et al., 2021). Effluents having concentrations of nitrogen as high as 20–100 mg/L trigger eutrophication risks, and microalgae vacuum up this nitrogen via uptake into proteins, which are 70% nitrogen biomass, and membranes. *Chlorella vulgaris* lead with around 1–2-day doublings and records of stripping about 90–100% of NH4-N, 80–95% PO4 from sewage streams, while working efficiently at 20–30 °C and pH 6–9. The biomass yields are about 1–2 g/L (Gupta et al., 2024). While Scenedesmus sp. forms grazers resisting colonies, and in line with *Chlorella’s* nutrient pulling, they pile around 0.5–1.5 g/L solids in piggery wastes. *Nannochloropsis* sp. is marine-adapted and can pack moderate percentages of around 30–60% of lipids, not limited to highly polluted effluents but also in diluted streams. Thus, making them ideal for simultaneous cleanup with 70% COD drop and biodiesel feedstocks. The integration of such species into treatment systems offers a sustainable and eco-friendly approach to water purification, replacing conventional chemical or energy-intensive methods (Khoo et al., 2020). Figure 1 shows some commonly used Microalgal species in biodiesel production.

**Figure 1:**
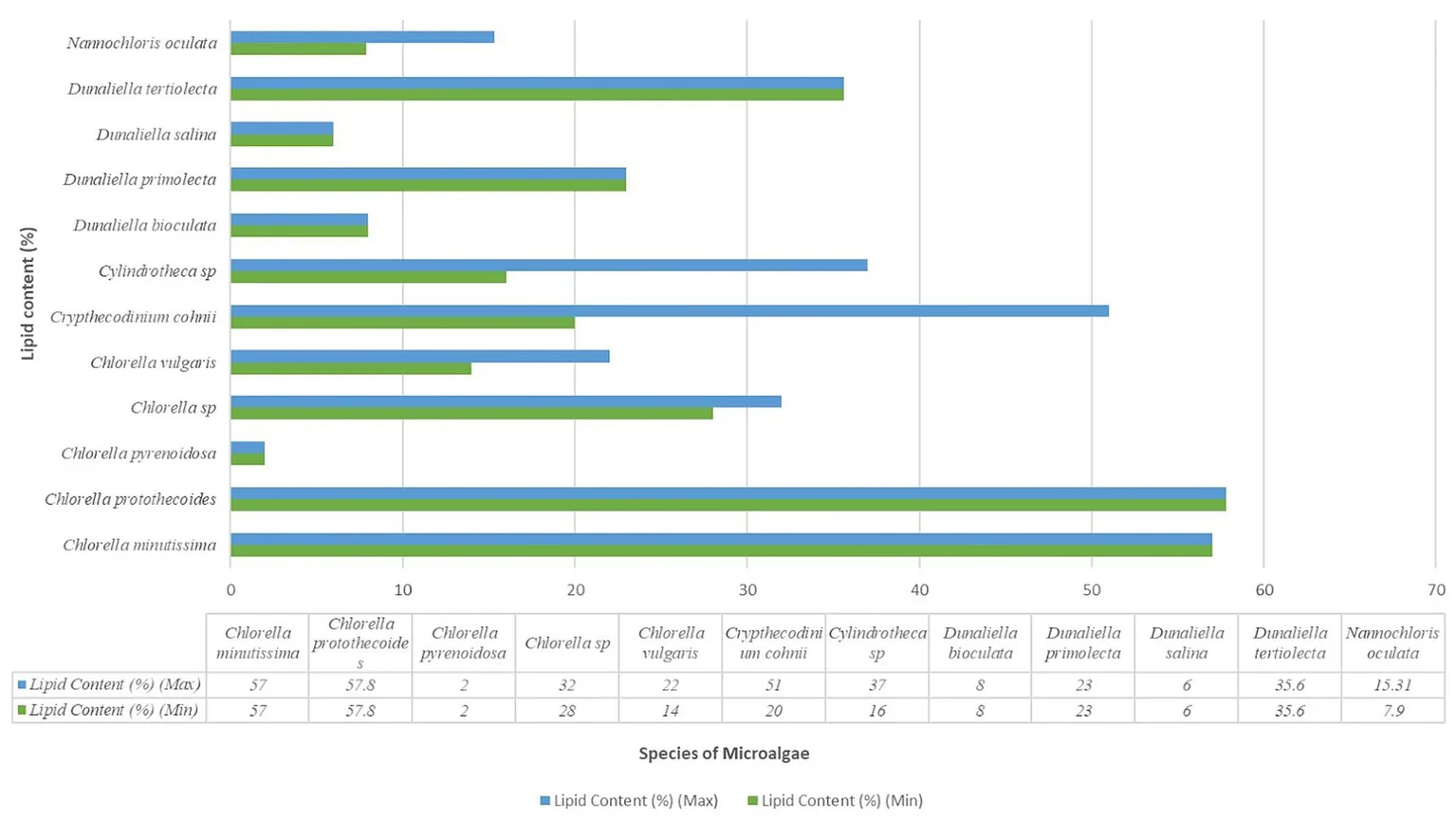
Common microalgal species used in biodiesel production.

The microalgae cells consist of bilayer membranes, encapsulating chloroplasts, Golgi body for secretion and vacuoles, and green algae have rigid walls made up of cellulose (about 2–5% dry weight) for structural support, and silica in diatoms as well, all aiding sedimentation in ponds (Dolganyuk et al., 2020). Presence of flagella is responsible to stir cultures naturally and they boost mass transfer by 15–25%; and *Scenedesmus* like colonies are formed, making it easier to harvest by flocculation through self-aggregation (Morales et al., 2021). A schematic cell structure for easier visualisation is shown in Figure 2. When talking about lipid accumulation by these unicellular microalgae (Zhu et al., 2022), lipids, triacylglycerols (TAGs) form droplet clusters of 5–20 μm in size in absence of nitrogen. Table 1 gives an overview of the percentage of lipids and biodiesel along with the heterogenous catalyst used for sustainable biodiesel production. The cells stop dividing and reroute carbon from carbs to fats through acetyl-CoA pathways. This contributes to around 40–70% of the dry weight of which 10–20% is vegetative. Larger cells like Chlorella (10–20 μm) can provide twice as much storage volume and surface tweaks like glycoproteins improve nutrient diffusion by almost 30%. Light about 100–200 μmol/m^2^/s, Nitrogen < 5 mg/L, are both stress gradients that can significantly trigger jumps in lipid by 2–5 times in over 3–7 days, however this must be balanced with C: N: P ratios (20:10:1) to avoid a fall (Shehhi et al., 2025).

**Figure 2:**
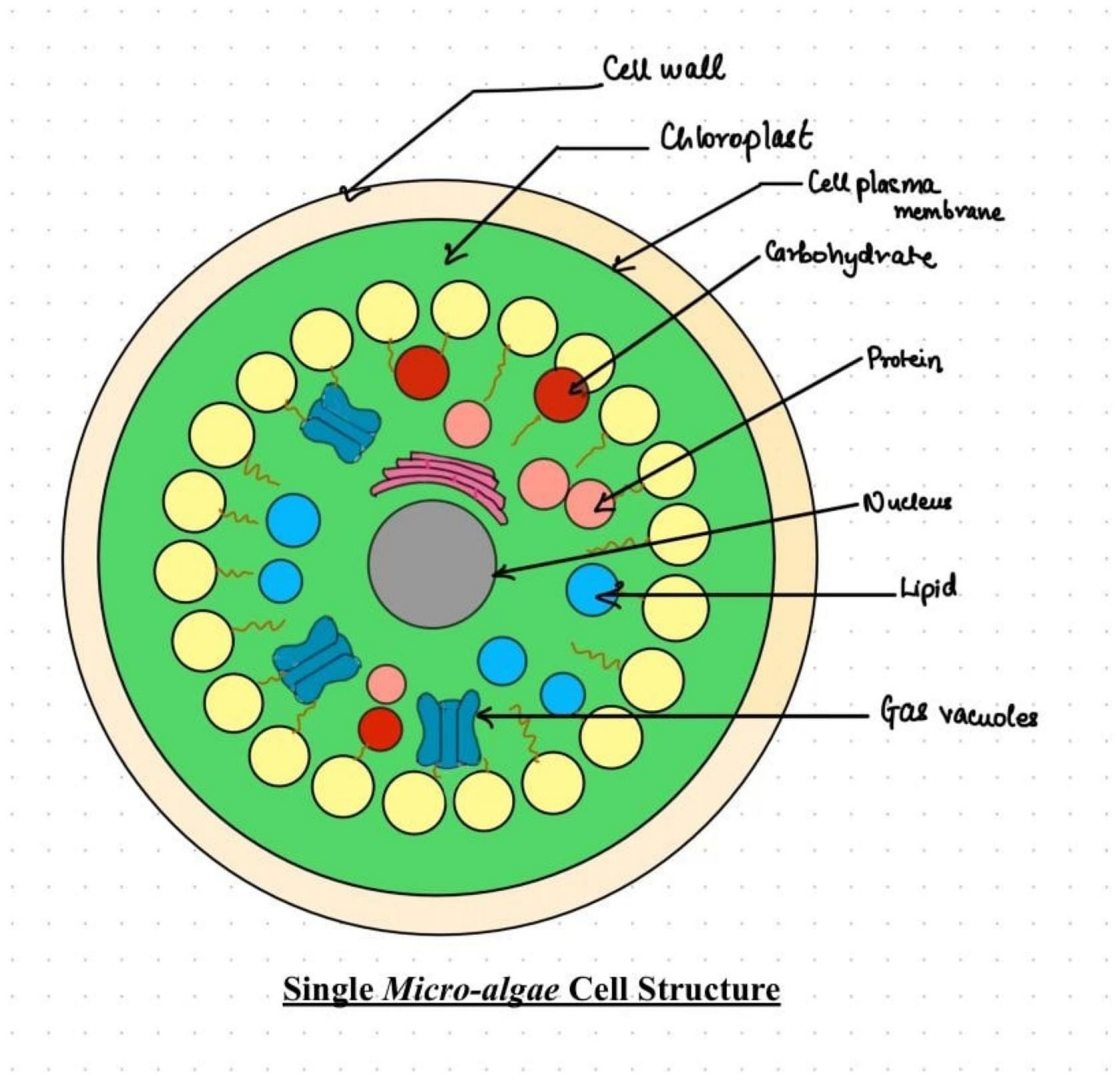
Schematic representation of Single microalgal cell structure.

**Table 1:** The percentage of lipids, catalyst used and biodiesel for certain type of microalgae.

Species of microalgae	Lipid content (% dry weight)	Catalyst used	Biodiesel %	Reference
*Nannochloropsis* sp.	40–60	Zn Al layered double hydroxide	95.2	Atashkar and Saidi (2024)
*Monoraphidium* sp. NCIM 5585	47.3 ± 0.02	CaO Bio- Nanocatalysts of residual biomass	86.5 ± 0.65	Pandey et al. (2025)
*Spirogyra* sp.	-	Phyco-synthesized CuZnO catalyst	91.05	Pourhoseini et al. (2026)
*Nannochloropsis oculata*	26.2	-	96	Askari et al. (2022)
*Chlorella pyrenoidosa*	-	Sulphuric acid	92.5	Cao et al. (2013)
*Botryococcus braunii*	26.3	Sulphuric acid	80	Hidalgo et al. (2015)
*Chlorella pyrenoidosa*	47.2% in 0.3% salinity	-	91	Huang et al. (2025)
*Chlorella Minutissima*	40–45	Sulfated carbonaceous catalyst	72.8	Arora et al. (2024)

### 1.1 Genetic engineering and life cycle sustainability in microalgae

Microalgae are rapidly growing photosynthetic organisms in the sense that some strains in them can increase their mass multiple times a day (Masojídek et al., 2021). The production of a range of products like lipids, hydrocarbons and complex oils (depending on the microalgal species) leads for more possibilities to explore the economic feasibility, and its improvement (Morales et al., 2021). To improve the economic feasibility of microalgal biofuels, significant research is focused on biotechnology and strain improvement. Genetic engineering techniques are being applied to enhance biofuel properties, such as by modifying lipid pathways to increase the production of triacylglycerols (TAGs), the primary feedstock for biodiesel (Khoo et al., 2023). Multiplying every 12–24 h in optimum conditions, select strains naturally can produce species-specific lipids like C16-18 chains of fatty acids, also some hydrocarbons like botryococcenes, or exopolysaccharides enhance the economic viability to scale them up commercially. CRISPR/Cas9 and alike techniques focus on repressors of β-oxidation, in order to prevent lipid breakdown in order to increase TAG synthesis, all in all, achieving much higher yields. This is seen in genetically engineered Chlorella strains, which reach around 60% lipid content in wastewater cultures, while RNA interference can silence catabolic genes for an additional 30% lipid gain (Saha et al., 2025). The utilization of genetically engineering microalgae can create many concerns regarding regulatory, biosafety and environment concerns. Any accidental evolution of modified strains into the natural habitat can lead to ecological competitions between microorganisms and cause consequences. Therefore, if we want to apply genetically modified microalgae for large scale application, it is necessary to employ risk assessment strategies and constant monitoring. GMO can be physically contained through closed or even semi close photobioreactors to help reduce the possibilities for the environmental release of the strain including engineered auxotrophy and safety mechanisms like kill switches (Čelešnik et al., 2016; Calatrava et al., 2024).

Life cycle assessment (LCA) can be utilized as a tool used to evaluate the complete environmental footprints. It reveals open raceway ponds have less energy requirement (1–2 MJ/kg biomass), when compared to PBRs (5–10 MJ/kg), however, hybrid systems can help in reducing eutrophication risks by 40% through recycling of effluents (Herrera et al., 2020). Meanwhile, the complete chains of production release up to 0.5–2 kg of CO₂ equivalents per kg of biodiesel and this is very less compared to fossil diesel’s 3.5 kg, with drying accounting for 30% of the impacts. Advantages specific to location, for example, India’s high solar irradiance lowers energy demands by around 20%, along with multiple product valorisations like fuels and proteins. All of this also reduces the potentials for global warming by 70%, with enhanced technologies and solutions like CO₂ or UV dosing in high-rate algal ponds (HRAPs), which improve nitrogen and phosphorus removal by as high as 85–95%, like observed in Brazilian field trials (Moradiya and Marathe, 2023).

### 1.2 Challenges and future perspectives

There are 4 generations of feedstock that has been studied for utilization of biofuel production. The different types of feedstocks along with their advantage and disadvantage are represented in Figure 3. It is true that microalgae technologies are promising in the integrated wastewater remediation biodiesel production systems, however, economic and technical hurdles continue to exist and block a large-scale adaptation or rollout (Srimongkol et al., 2022). Microalgae are known to outperform traditional crops in production of biodiesel, offering 20–50% lipid content, 10–50 times faster growth, also they have no farmland competition, making it ideal for wastewater-based systems (Zhang et al., 2022). With high production costs leading at $4-10/kg biomass, it is driven by energy intensive cultivation like use of paddlewheels, aeration etc. adding to 40–60% of shares, harvesting with centrifugation and lastly, extraction with solvents etc- all in all, totaling to $2-6/L biodiesel versus fossil diesel’s $0.8/L (Maroušek et al., 2023). Breaking it down to scalability challenges, these arise scaling lab experiments in big outdoor ponds, where microalgae suffer from zooplankton grazing (20–50% loss), sunlight is blocked from deep ends and weather changes like monsoons lead to declining outputs significantly (Kanwal et al., 2025; Casanova et al., 2022). Despite all the progress achieved till now for microalgal biodiesel production, the main challenge is to make this process commercially available. At industrial scale the maintenance of high biomass cultivation and harvesting along with lipid extraction becomes difficult because of the variations in temperature, intensity of light, nutrient concentrations, contamination risks and sufficient CO_2_ transfer. If we use open pond system of high depth then it’s possible that the light will not penetrate till the bottom resulting in low biomass productivity. Therefore, there is an immediate need of cultivation system that can maintain good biomass productivity even with little fluctuations in environmental conditions (Semwal and Joshi, 2026). For heterogenous catalysts, the commercialization needs specialized optimization for the synthesis pathway, structural stability, catalytic activity and its reusability. Catalysts made of waste animal bones, oyster shells, egg shells can make the process economically efficient but large-scale applications can be affected by acquiring and transportation, synthesis, reproducibility, leaching of elements and recovery. So, therefore it is important to develop stable catalysts supported with MgO, ZrO₂-supported catalysts, sulfonated carbon materials to increase activity and stop catalyst to move toward deactivation. Nowadays many nano-catalysts are also being produced for better stability. Addressing these technological and scientific challenges will help improve reliability and efficiency for large scale process (Mandari and Devarai, 2021). Now, although wastewater works well as a nutrient source, most times it gets thrown off due to uneven pollution-pollutant levels. Next, the issue of the absence of standardization puts a significant hurdle in the process, stalling them. There are no standard rules or global benchmarks for strain purity (>95% monoculture), or media consistency (C:N:P ratios) (Magyar et al., 2023), or biodiesel quality standards being met, plus missing laws for GMO tests and water reuse policies, all contributing to the list of challenges (Akram et al., 2025).

**Figure 3:**
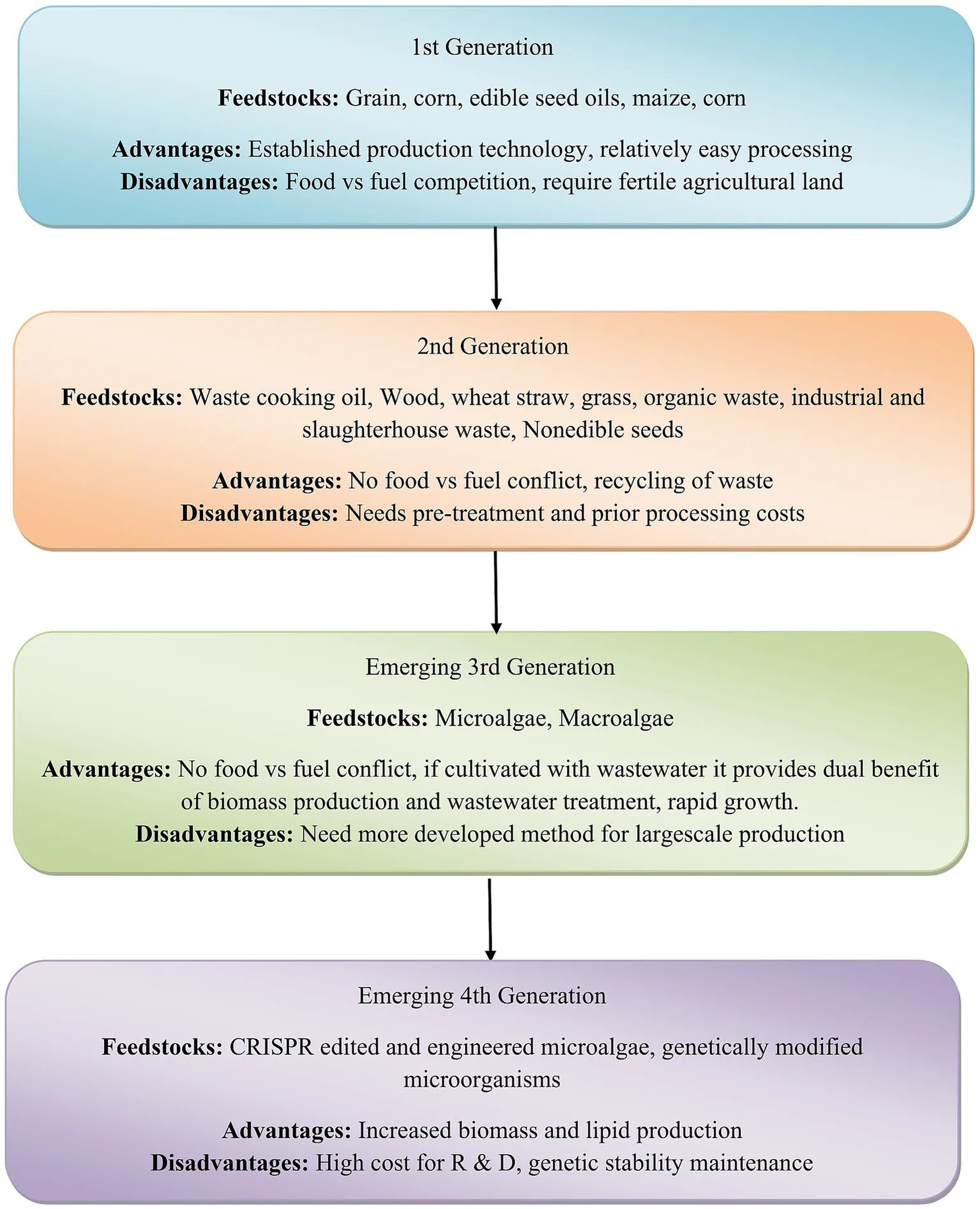
Different types of biodiesel feedstocks and their pros and cons.

Therefore, future fixes consist of utilization of wastewater for free nutrient intake, saving major costs and supporting remediation greatly, and special solid catalysts which can be repurposed more than once, allowing making fuel more economically accessible without increasing investments (Orege et al., 2022). Microalgae sequester over 1.8 kg CO₂/kg biomass from flue gases and this offsets emissions by 50–90% in cofiring setups. The biomass remaining after lipid extraction is rich in proteins and carbohydrates and can be processed into animal feed, biofertilizers, or other valuable bioproducts, ensuring that almost all of the biomass is used. The various applications of microalgae are elaborated in Table 2. Not limited to 20–50% lipids for biodiesel production with FAME via transesterification giving about 90% yields, the leftover residues have been known to furnish 40–60% proteins for fishmeal or aquaculture feeds, and pigments (cartenoids) as well as 10–30% carbs for biogas. Secondary metabolites like polyunsaturated fatty acids (PUFAs), and sulfated polysaccharides, also have applications in the pharmaceutical, nutraceutical, and food industries (Mata et al., 2009).

**Table 2:** Summary of various applications of microalgae in health, industrial and environmental sectors.

Microalgae application	Health benefits	Industrial applications	Environmental applications
Antioxidants	Polyphenols, carotenoids, vitamins	Pharmaceutical drug development	Carbon sequestration (CO_2_ capture)
Antitumor activity	Carotenoids, polysaccharides	Nutraceuticals, functional foods	Bioremediation (pollutant removal)
Antimicrobial	Peptides, sterols, alkaloids, amides	Skincare, personal care products	Wastewater treatment
Anti-inflammatory	Carotenoids, carbohydrates, PUFAs	Dietary supplements, food additives	Bioremediation
Antiviral	Sulfated polysaccharides, carotenoids	Animal feed, aquaculture	Biomonitoring of water quality
Uv protection	Pigments (carotenoids, phycocyanobilins, phycoerythobilins)	Algae-based bio-fertilizers, soil amendments	UV filters
Pharmaceuticals	Polyphenols, carotenoids, peptides	Pigments, bioplastics, biopolymers, nanoparticles	Biodegradable Waste treatment
Nutraceuticals	Proteins, omega-3 fatty acids, vitamins, minerals	Food, pharmaceuticals, and agriculture	-
Food industry	Proteins, omega-3 fatty acids, vitamins, minerals	Thickeners, Emulsifiers	-

Smart AI integration and AI driven controls like RL for DO or pH, can boost yields by 30–50%, as well as hybrid PBR-ponds and advanced electro-coagulation harvesting techniques can boost growth too. Using iron-oxide nanoparticles in form of magnetic harvesting has also been known to improve harvested yields (Markeb et al., 2019). Test plants aim for affordable fuel solutions by 2030 with the support of the government (Wu et al., 2025).

## 2 Microalgae cultivation techniques

Producing biomass is an important and key step in any microalgae biotech for wastewater treatment and biofuel products. Cultivation techniques or methods are divided by their open or closed feature, and by method of nutrition, like (photoautotrophic via wastewater/sunlight, mixotrophic with organics).

### 2.1 Basic growth conditions

Macronutrients like nitrates (10–50 mg/L), phosphates (1–10 mg/L), and ammonium are essential for photosynthetic microalgae (Dolganyuk et al., 2020) for proteins and cell division. Wastewater being rich in these, naturally provide these to the microalgae and end up reducing the synthetic media requirements for growth by 70–90% (Abdelfattah et al., 2022). Although, in case of fresh nutrient requirements can lead to increased production costs as well (Yu et al., 2022). BG-11 is an excellent media with promising yields of 1.1–3 g/L for *Chlorella vulgaris* with high levels of chlorophyll (20–30 μg/mL), with carbs (30–40% dry weight), especially in cases with low nitrogen concentrations, where it proves better than other media by almost twice as much oil production (Pandey, Soni et al., 2023). The most optimal pH conditions are in the range of 6.5–8.5 (although green algae can handle 4–10), and this is naturally steady from the usage of carbon dioxide but needs buffers in highly alkaline effluents with >9 pH. Ideal temperature (Song et al., 2024) is 25–30 °C and it maximizes the growth rates with doubling every day or two. However, over 35 °C leads to loss in output level by half as it is responsible for enzyme denaturation (Zittelli et al., 2022). Light intensity requirement is specifically moderate (100–300 units) with 12 h- light and 12-h dark period, in order to protect against any photoinhibition damages from the sun as >1,000 units harms energy centres in the cells. Lastly, wastewater’s turbidity is responsible for even distribution of the light (Rahman and Hellgardt, 2025).

### 2.2 Overview of large-scale cultivation techniques

Open raceway ponds (ORPs) are the cheapest and most common methods for large-scale microalgae cultivation. We can utilize the wastewater for growing microalgae in open pond system. Photobioreactors (PBRs) are more specified and sophisticated and contains many probes for monitoring growth, temperature, pressure and dissolved oxygen. It has baffles fixed at the walls of the reactor so the cells do not get much pressure and premature cell breakage does not happen. The bioreactor has inlet and outlet pipes for addition of media and removal of any gas or foam, respectively. It is also equipped with machine that helps with reduction of saponification. This continuous monitoring results in greater amount of biomass production in less time. The ring sparger in the bottom helps with the aeration by releasing oxygen inside the chamber for the growth of microalgae. The size of the sparger can be changed according to the size of the air bubble required (Biswas et al., 2025). The detailed diagram of a typical PBR is represented in Figure 4.

**Figure 4:**
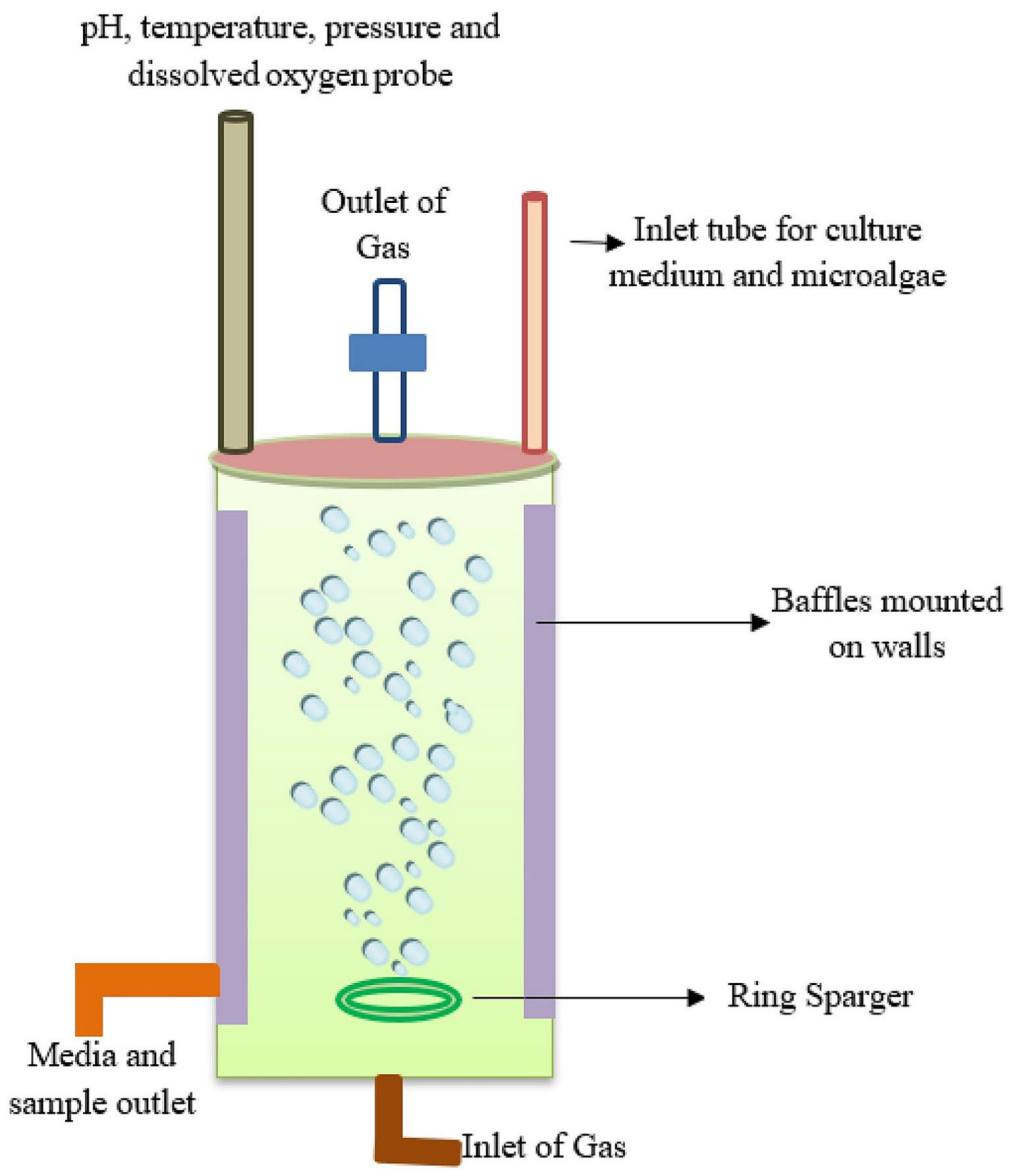
Detailed diagram of photobioreactor (PBR).

ORPs costs range between $0.1–0.3 per square meter to build and $0.02–0.05 per kg of biomass. Paddlewheels are used for gentle stirring and circulation at slow speeds, in shallow ponds (0.20–0.40 m in depth). They work advantageously when using wastewater to remove around 70–90% of phosphorus and nitrogen. The construction and operational ease and scalability back them up to be a successful option, however, the rate of evaporative losses of water (5–10%) on a daily account and contamination crashes in crops from bacteria/rotifers, along with uneven mixing causing gradients, all are significant drawbacks of ORPs (Debnath et al., 2025). On the other hand, closed PBRs inculcate clear tubes (5–10 cm wide) or flat panels (2–5 cm gaps) for total control. This helps deliver 2–5 times more biomass (10–20 g/m^2^/day), block contamination and adjust or fine-tuning O₂/CO₂ levels with air bubbles. Although, PBRs are cost intensive and have high capex ($50-200/m^2^), prone to fouling, causing 10–20% productivity drop and also have scaling limitations on sizes (Skifa et al., 2024). Hybrids with starting algae cleanly in PBR and growing in ORP (bulk, cheap ponds) can lead to balanced, lower costs with wastewater and good yields (1–2 g/L) (Velásquez-Orta et al., 2024).

When investigating CO_2_ capture, industries like powerplants that release CO_2_- rich flue gases are looked at, and these gases are which the microalgae use as food for growth in PBRs or photobioreactors (Veerabadhran et al., 2021). A PBR facility, Italian and industrial-scaled, coupled flue gas to semi-continuous culture and resulted in productivity boost, cutting greenhouse gases as well as nutrient recovery, although electricity requirement for cultivation still contributed as a key cost (Penloglou et al., 2024). This integration also supports circular bioeconomy by reusing industrial emissions. Microalgal biorefineries (Geng et al., 2025) process algae into multiple products like biodiesel, biogas as well as nutraceuticals after cultivation. Latest discoveries and advances include hybrid systems with open ponds or PBRs (Nneoma et al., 2025), genetic tweaks in order to achieve better yields, wastewater co-use and AI monitoring, all with aims to lower costs and energy use (Kanwal et al., 2025). As an example, full value chains cover cultivation and harvesting, both by low-energy methods like flocculation, and conversion. The production of these valuable co-products boosts economic sustainability. Moreover, it has been observed that prototypes associated with waste streams are capable of scaling up effectively. Figure 5 provides an overview of the potential benefits for real world applications.

**Figure 5:**
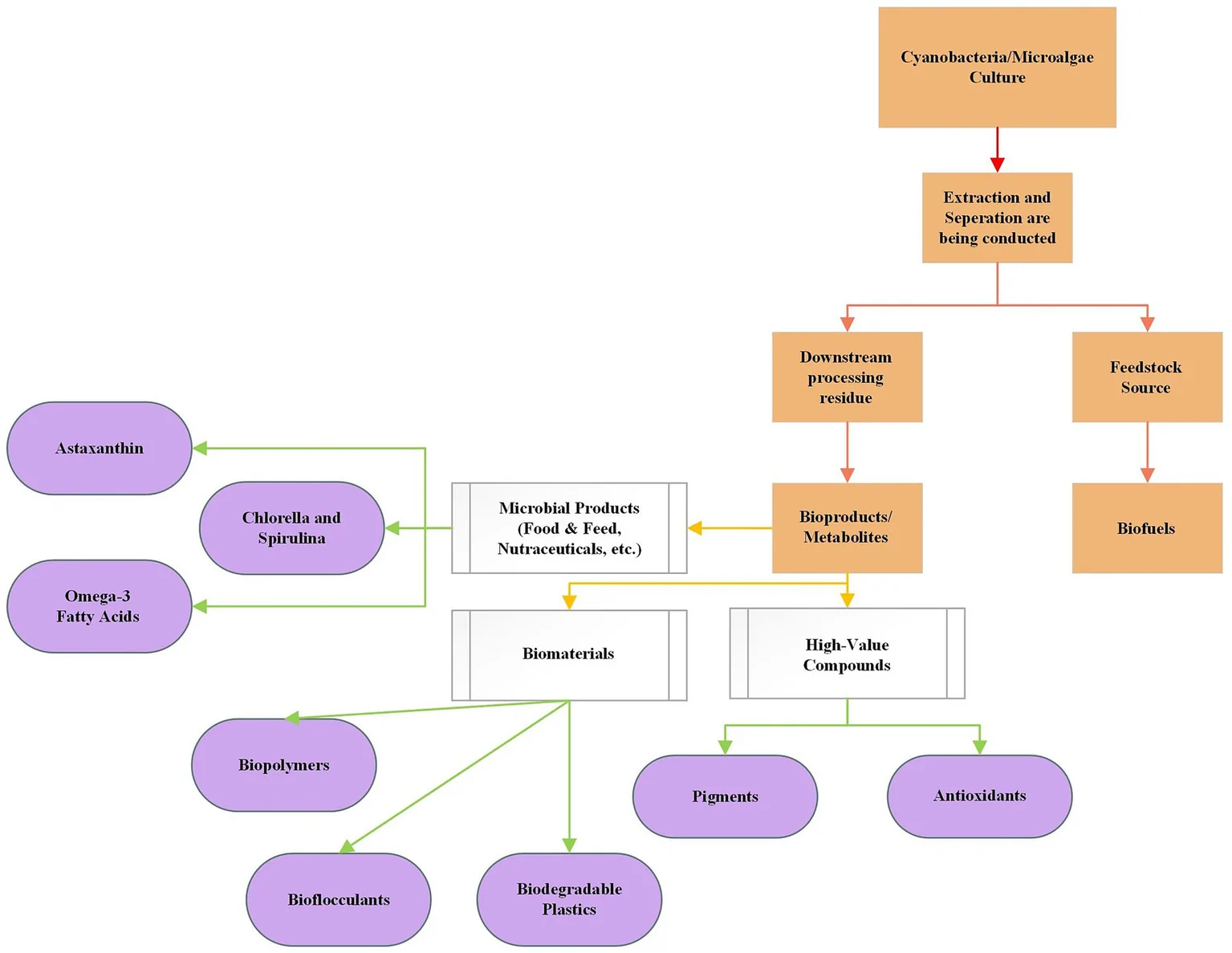
Schematic representation of potential benefits for real world applications.

### 2.3 Cultivation of microalgae using waste water integrated system

The treatment of industrial and domestic origin wastewater is a crucial aspect of environmental management. Industries generate a growing amount of wastewater, which contains contaminants that are harmful to ecosystems and public health (Silva, 2023; Savari et al., 2025). Various research and studies have backed the claims that microalgae can be used to clean wastewater from industries, farms or homes by the process of absorbing nutrients and pollutants present in the sources of collected wastewater. This happens while growing biomass, which has potential to get converted into fuels or feed (Singh et al., 2023). The processes involved can take place in open-ponds or PBRs, as well as in hybrid systems, which have proven to outperform traditional methods by handling nitrogen, carbon and phosphorus in a single step (Kundu et al., 2024). For example, microalgae systems remove 45–65% of BOD (biochemical oxygen demand) and COD (chemical oxygen demand), plus they naturally disinfect water and cut secondary pollution (Dammak et al., 2023). Clear comparisons between treatments using and not using microalgae are shown in Figure 6.

**Figure 6:**
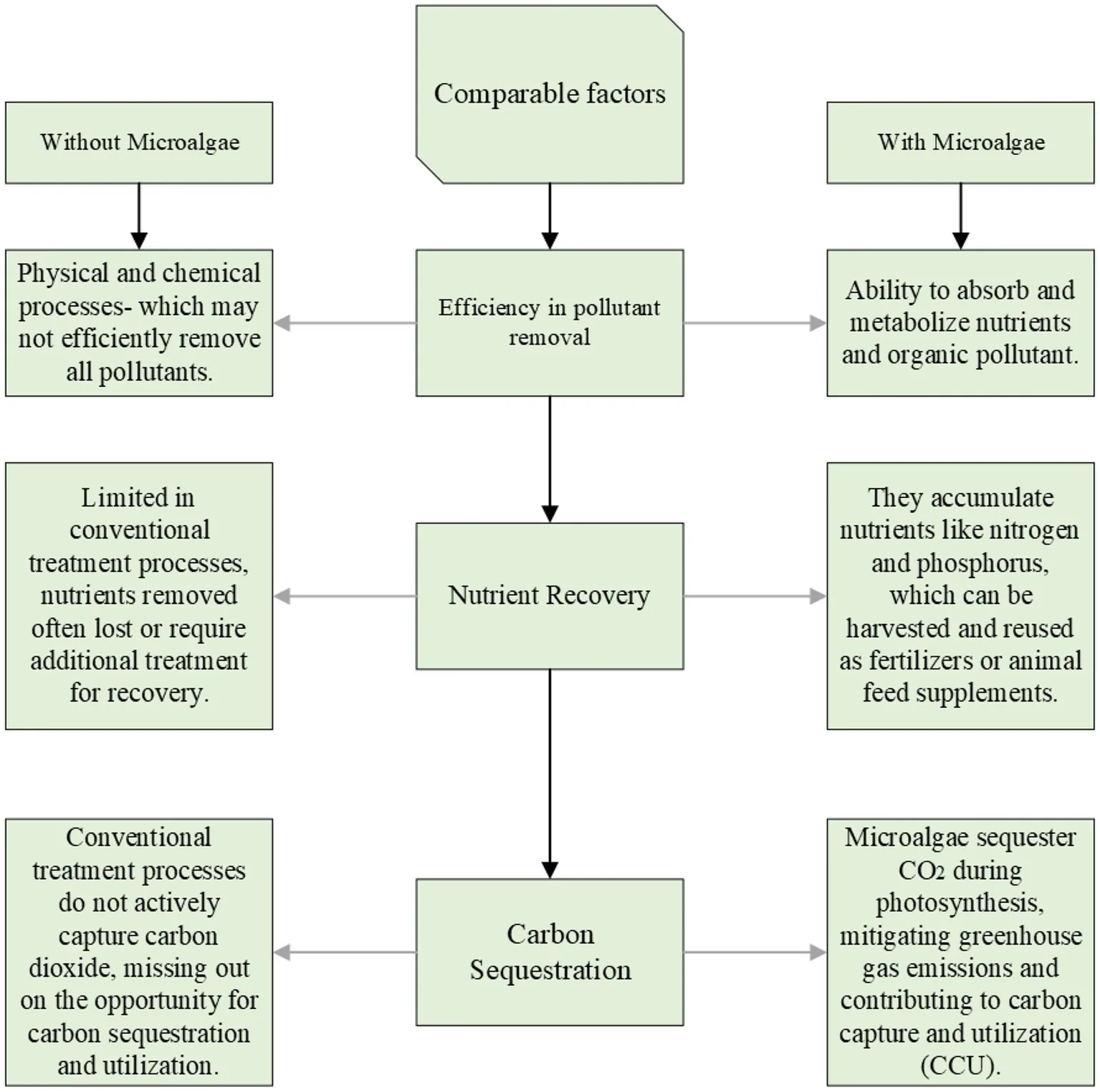
Comparison of treatment with and without microalgae.

There are several simple steps for microalgae cleaning water. One is biosorption, where pollutants stick to the algae’s outer layer like magnets (Fernández et al., 2024) (with up to 90% removal for dyes like Reactive Red 120), other is bioaccumulation, which pulls toxins inside cells (around 50–80% for metals like lead) (Chan et al., 2024) and then, biodegradation using enzymes to consume organics with about COD reduction of 70–95% in 5–10 days (Mustafa et al., 2021). Photosynthesis takes up CO2 and pumps out oxygen for helpful bacteria and the pH rises to 9 to 11 and causes metals to clump and eventually sink out of water. *Chlorella vulgaris* has proven to remove 95% ammonia nitrogen from piggery wastewater (NH4-N 100 mg/L to 5 mg/L), and 90% phosphate (PO4-P from about 20 mg/L to 2 mg/L) in batch cultures, along with 1.2 g/L biomass (Flores-Zambrano et al., 2025). With optimal temperature about 20–30 °C, and light 5,000–10,000 lux, pH 7 to 9, the process becomes concentric, hence making it reliable for real-world wastes like urban sewage or farm runoffs, whereas for biosorption particularly, pH varies from 3.0 to 6.5 for metals and microalgae species (Abdelfattah et al., 2022). Microalgae are also known to act like sponges for toxic heavy metals in industrial waste, and using cell walls rich in proteins and carbs for binding ions via electrostatic forces, can be observed by FTIR, OH group peaks at particular 3,400 cm^−1^ (Spain et al., 2021). In lab studies at pH 6–8, *Chlorella sorokiniana* removed 98% lead (Pb from 10 mg/L), 95% cadmium (Cd), and 92% copper (Cu); green biofilms in mine drainage removed 100% Cu and Al (initially 20–50 mg/L), 99% Cd, 95% Zn, and 85% Fe during 5 days of continuous flow (Kazakis, 2025). *Scenedesmus obliquus* tolerated 85–99% chromium (VI) (Daneshvar et al., 2019), (5–50 mg/L initial) with a maximum capacity of 120 mg/g dry weight, flourishing at 1–5 g/L dosage and 24–120 h contact time. The benefits include elimination of sludge buildup, the ability to reuse loaded biomass for biofuels, and tolerance to concentrations of 1–100 mg/L, which is superior to chemical precipitants that cost 2–5 times more (Pradhan et al., 2022).

#### 2.3.1 Phycoremediation applications

Phycoremediation is the use of microalgae to fix polluted water (Singh et al., 2016), soil, or air from various nutrients, dyes, metals, organics, etc. and it’s often termed as the “green cleanup” (Pandey, Narayanan et al., 2023; Pradhan et al., 2022). It’s the eco-friendly process (Sunday, 2018) that produces oxygen and biomass around 0.5 to 2 g/L-day, meanwhile reducing the costs to $0.5–2 per m^3^ compared to $5–10 for activated sludge. It is very industry-friendly (Sarma et al., 2021), specifically for mining or pharma domains, with scalability for ponds (up to 1,000 m^2^) or reactors, with Chlorophyta (e.g., Chlorella) (Wang et al., 2023) leading due to fast growth (doubling in 24 h). Table 3 below gives a comparative view of various industrial applications of Phycoremediation.

**Table 3:** Comparison of various industrial phycoremediation applications.

Industry	Microalgae used	Key pollutants	Removal efficiency	Advantages	References
Paper & Pulp	*Chlorella, Scenedesmus*	BOD, COD, metals	77–88% COD/N/P	90% lower costs	Koul et al. (2021); Walters et al. (2024); Sharma et al. (2020)
Distillery	*Scenedesmus* sp.	COD, phenolics	70–95%	70% energy savings	Walters et al. (2024)
Mining AMD	*Scenedesmus*	Metals, acidity	85–98% metals	No chemicals	Kumar et al. (2024); Walters et al. (2024)
Petrochemical	*Chlorella vulgaris*	Phenol, hydrocarbons	90–98%	Toxin tolerant	Das and Deka (2019); El-Gendy and Nassar (2021)
Textile/Pharma	*Chlorella strains*	Dyes, antibiotics	95–99%	Fast biomass growth	Talib et al. (2023)

There are various and a number of uses for microalgae-based wastewater treatment in different sectors like paper and pulp manufacturing, petrochemical, food and beverage manufacturing as well as pharmaceuticals (Abdelfattah et al., 2022). Different sectors have different effluent discharges (Goh et al., 2022). In the food and beverage sector, microalgae are employed to clean wastewater streams that include pollutants from processing operations, nutrient contents and organic matter. This is helpful in guaranteeing adherence to environmental laws and regulations by significantly and efficiently eliminating pollutants. This involves lowering chemical oxygen demand (COD) and biochemical oxygen demand (BOD) levels as well (Młyńska et al., 2024). Lignin, colorants and organic compounds are similarly eliminated from effluent streams of paper manufacturing processes in pulp and paper industries, by integration of microalgae-based systems (Baghel et al., 2020). This also includes creating algae-growing setups that best provide nutrients and ideal conditions, plus selecting algae types that can break down tough organic waste. Likewise, the oil industry employs these algae methods to clean hydrocarbon-polluted wastewater via processes like natural breakdown and absorption. Such tailored steps ensure strong pollutant removal, protect the environment, and comply with regulations (Kermani et al., 2024).

Integration of waste water majorly involves growing tiny algae with the help of wastes from factories or some treatment plants (Gurreri et al., 2024), where the pollutants are turned into useful biomass for fuels, feeds or chemicals. This has been proven to be an effective contributor toward circular economy, and creates valuable products, cuts costs as well as treats waste. Recent studies have shown various practical ways of undertaking this, such as linking algae growth to carbon dioxide capture or wastewater cleaning. Focusing on wastewater treatment (Avila et al., 2021), we form the link as microalgae naturally absorb nutrients like phosphorus and nitrogen from industrial wastewater (Pacha-Herrera et al., 2021), like dairy or urban wastes, all the while growing biomass (Hajri et al., 2024). A set up with microalgae-bacteria mix compared to using fresh water with fertilizers, the 80 m^2^ raceway reactor produced harvestable algae and removed ammonia from municipal effluent (Morillas-España et al., 2024). Another technique harvests microalgae rapidly using activated sewage sludge; at a 1:4 sludge-to-algae ratio (Tiwari and Prajapati, 2025), it reaches 100% harvesting in 8.5 h. The mixture is subsequently co-digested to produce more biogas (382 mL/g VSS) than either algae or sludge alone.

## 3 Lipid extraction of microalgae

When focused mainly on lipid extraction from microalgae, the aim or purpose is biodiesel production. Apart from targeted lipids and sample types, the factors affecting the choice of technique employed for lipid extraction are also efficiency, safety and costs. There are broadly two categories for extraction approaches: conventional and mechanical methods. To begin with, the conventional method of lipid extraction, the prime solvent mixture, consists of polar and non-polar solvents, which is done in order to cover a variety of lipids targeted. The famously employed methods with this mechanism include the Bligh and Dyer method and the Folch Method. Meanwhile, the mechanical or the “non-solvent” method is a more sustainable and environmentally friendly approach. A study focuses on the different methods for the current microalgae lipid extraction, and a comparative study identifies that Soxhlet extraction showed that a mixture of hexane and ethanol yielded nearly three times more lipids than using hexane alone, demonstrating the efficiency of using mixed solvents (Li et al., 2014; Behera et al., 2021). Another study that focused on the pretreatment methods in the laboratory for biodiesel production from microalgae brings to light the bead milling method which uses methanol to give a lipid recovery of approximately 24% (wt%), while without it, higher recoveries of over 70% were obtained at higher temperature conditions (Kumar et al., 2016). A research comparing the source to their lipid contents related the lipid yield with varied species through experimentation, making it relevant and backing up its optimisation. The data points toward a significantly wide range of natural lipid content across species, such as 23–68% of dry weight for *Nannochloropsis oceanica* and 9–17% for *Euglena gracilis*. Some environmental factors also play a critical role in the lipid yield improvement, as per the study, stating a 5% CO₂ concentration increases the lipid content in one species to 65% and a nitrogen-to-phosphorus ratio reduction increases yield to 44.38% in another. So, it can be concluded that while species selection is a prominent factor, so are the cultivation conditions for an optimized yield (Xin et al., 2024).

### 3.1 Comparisons and analysis of different lipid extraction methods

As initially described, the Bligh and Dyer method is applicable for a chloroform–methanol–water ratio of 1:2:0.8. The Bligh and Dyer method is favourable as a much lower solvent-to-sample ratio (1:3) is required as compared to other methods making it more profitable and high lipid recovery yields (Amna and Faheem, 2025; Saini et al., 2021). In a study, the conventional Bligh and Dyer solvent mixture (methanol: chloroform) showcased the highest total lipid recoveries at 100 °C. The results found were 352 ± 28 mg/g for *Chlamydomonas reinhardtii*, 410 ± 32 mg/g for *Chlorella vulgaris*, and 127 ± 15 mg/g for *Arthrospira platensis*. This proves that there is high effectiveness for this method under regular growth conditions. Even for microalgae under low-nitrogen growth conditions, the highest lipid recoveries at 100 °C, with 550 ± 21 mg/g in *C. reinhardtii* and 612 ± 29 mg/g in *C. vulgaris* were found from using the Bligh and Dyer method. This highlights its effectiveness even when neutral lipid accumulation is high (De Bhowmick et al., 2024). As the Bligh and Dyer method works well for polar lipids, it was found that the extraction of both polar and non-polar lipids was highly effective by combining the ultrasound extraction technique. The yield of n-alkanes is almost double compared to Bligh and Dyer Ultrasound Extraction, or Accelerated Solvent Extraction individually, when this combination is used as BDE alone might give a lower estimate of non-polar lipids (Zhou et al., 2023). The Folch method plays a vital role in extracting lipids from different biological samples mixed in a 2:1 volumetric ratio using chloroform and water. It’s known to dissolve almost all types of lipids with a significant efficiency (Saini et al., 2021; Omar and Zhang, 2023). However, this method is validated and reproducible for various biological samples and is effective for extracting a wide range of lipid including phospholipids and triglycerides. Moreover, recent studies have been striving to improve the method by making it safer with less toxic solvents, quicker and more adaptable with increased lipid extraction efficiency. While there are some other comparative studies between the Folch method and other extraction techniques like Bligh and Dyer or MTBE (methyl-tert-butyl ether) and other newer green solvent methods, Folch remains to be the gold standard for thorough lipid extraction from microalgae. To improve the technique with speed and efficiency, single-step adaptations have been explored for microalgal lipid screening processes (Wittenhofer et al., 2024).

## 4 Biodiesel production utilizing photosynthetic microalgae

Fatty Acid Methyl Ester (FAMEs) as it’s discussed earlier are a part of the biodiesel, and serve as the key components responsible for fuel stability, clean burning, as well as being suitable for engine uses. When a FAME profile is carried out, it lists out the types and amounts of fats like different chain lengths in percentages (C16:0, C18:1), and with an optimal mix (Maes et al., 2023), the focus is on saturated fats (20–40%, e.g., palmitic acid C16:0) for oxidative stability as fuel can stay fresh for longer duration (more than 8 h achieved in tests), without gumming. On the other hand, monounsaturated fats (30–50%, e.g., oleic acid C18:1) are best suited for good cold-flow properties (Silva et al., 2023) like staying in a liquid state at −5 °C to 5 °C cloud point, while meeting standards too like ASTM D6751 or EN14214. Each algae species gives a unique profile. For example, *Scenedesmus quadricauda* (Wong et al., 2015) is known to show C16:0 around (25–35%), C18:1 (40–50%), and C18:2, dominantly, along with >85% FAMEs which are suitable, while being optimized by nitrate/phophate ratios in the growth medium. Also, *Chlorella pyrenoidosa* reaches upto 45% lipid yields when under nutrient stresses from wastewater and this causes shifts toward stable C16:0/C18:1 for improved storage and engine uses (Kirrolia et al., 2024). It is known that wastewater conditions can naturally favour fats of these qualities, unlike polyunsaturated fats which oxidize faster and cause clogging in colder conditions (Tu et al., 2016). Furthermore, Table 4 elaborates high lipid containing microalgal species including *Chlorella vulgaris*, *Scenedesmus* sp., *Nannochloropsis* sp., and *Chlorella pyrenoidosa* compares differences in their lipid content, major fatty acid composition, wastewater tolerance, phycoremediation potential, and scalability.

**Table 4:** Comparison of high lipid containing microalgal species based on biomass productivity, lipid content, fatty acid composition, wastewater tolerance, phycoremediation potential, and scalability for sustainable biodiesel production.

Species	Biomass productivity	Lipid content (% dry wt)	Major fatty acids (FAME profile)	Wastewater tolerance	Phycoremediation potential	Scalability
*Chlorella vulgaris*	High (1–2 g/L; fast growth; 1–2 days doubling)	High (40–60%)	C16:0, C18:1, C18:2	High	High (N, P, metal removal; >90%)	Good
*Scenedesmus* sp.	High (0.5–1.5 g/L in waste; colony-forming)	Moderate (20–40%, up to 47%)	C16:0, C18:1, C18:2	High	High (heavy metals, organic pollutants)	Good
Nannochloropsis sp.	Moderate (marine; 1–1.2 g/L typical)	High (30–60%)	C16:0, C16:1, C18:1, C20:5	Moderate-High	Moderate-High (COD/N removal)	Good
*Chlorella pyrenoidosa*	High (up to 47.2% lipids in saline stress)	High (up to 45–47%)	C16:0, C18:1	High	High (N, P removal in wastewater)	Good

### 4.1 Characterization of biodiesel

#### 4.1.1 Fourier transform infrared (FTIR) spectroscopy

FTIR spectroscopy offers a quick, non-destructive approach for analysing lipid composition in microalgal biomass by measuring characteristic vibrational absorption bands associated with lipid functional groups. In a study, FTIR spectra were able to distinguish triglycerides and phospholipids based on characteristic spectral patterns, primarily within the CH₂ and CH₃ stretching region (3,025–2,954 cm^−1^), the ester carbonyl region (1,746–1,654 cm^−1^) and phosphate- or hydroxyl-associated bands between 1,200–500 cm^−1^. In combination with chemometric modelling, FTIR made it possible to predict lipid content in microalgae which spiked across nine concentration levels up to 3% (w/w), indicating that this approach can detect minor variations in lipid composition using only small biomass quantities (Laurens and Wolfrum, 2010; Pasieczna-Patkowska et al., 2025).

#### 4.1.2 Scanning electron microscopy (SEM)

SEM aids in observing the surface level changes in microalgal cells post lipid extraction. Simultaneously, EDS supports this by identifying the elemental composition of the surface. This is done by analysing the X-rays emitted during the interaction of the electron beam with the sample, which is detected by the EDS to confirm specific elements. Previous studies on *Chlorella vulgaris* exposed to ZnO nanoparticles has proved that EDS mapping can clearly help in understanding material-cell interactions. Together, SEM with EDS is essential for detailed microalgal analysis (Covarrubias et al., 2025).

#### 4.1.3 X-ray diffraction (XRD)

XRD is used to analyse the structure of crystalline materials. XRD is a reliable technique used in the field of microalgal biodiesel production for material identification (Bunaciu et al., 2015). XRD is an important tool to understand the crystallinity and phase composition of different catalysts. A study on biodiesel production through transesterification of *Chlorella Vulgaris* revealed that XRD analysis showed distinct diffraction peaks confirming the successful conversion of CaCO_3_ by calcination into CaO nanoparticles used as the catalyst (Davoodbasha et al., 2021). Similar observation was seen in another study by (Pandey et al., 2025), where clear diffraction peaks at specific 2θ values was noted along with minor peaks confirming the crystalline nature of the CaO nano catalyst for biodiesel production from microalga *Monoraphidium* sp.

#### 4.1.4 Gas chromatography–mass spectrometry (GC–MS)

GC–MS is the widely used, standard tool for analyzing FAMEs. The samples undergo extraction with solvents like CH₃OH:CHCl₃:H₂O 2:1:0.8 and then undergo derivatization, where fats are vapourized using N, O-bis(trimethylsilyl)trifluoroacetamide, hence separating more than a hundred compounds in order of chain length, followed by identifying them by mass (even if in trace levels (ppm)). Testing for tropical microalgae *C. tenuissimus* using GC–MS and Nuclear Magnetic Resonance (NMR) techniques (Kock et al., 2019), 258 metabolites were found, with around 126 of them identified. This, in turn, proved that it can also spot low-concentration FAMEs and is critical for quality specifications like iodine levels <130 (due to fewer double bonds), along with more than 47, the value of cetane number, proving smoother ignition and, all in all, confirming a great, more than 90% success in lipid to FAME conversions (Alothman et al., 2024). GC–MS remains highly relevant even with the advances in technologies because it verifies that the fuel meets the standards, plus it guides various tweaks (for eg., low levels of N/P for more C18:1). Meanwhile, NMR compliments the function by detecting volatiles without destructions (Peng et al., 2023), whereas, multi-technique loops of GC–MS, NMR and FTIR together, simultaneously prepare a complete image with ester peaks (1740 cm^−1^), energy content (37–39 MJ/kg), and carbon-neutral scores (70–90% compared to fossil). This guarantees that the mechanism of bioremediation and biofuel production with wastewater-microalgae-biodiesel system, gives positive results in trucks or generators as well, at B20 blends without changes in the engines (Kumar et al., 2015).

### 4.2 Challenges and future directions in biofuel research

The entire technique of this form of bioremediation and biofuel production can be observed with a few key hurdles, but with smart fixes due to technological advances. To begin with the tricky growing conditions required by algae, optimum light, temperature about 25–30 °C and nutrients for absorption like nitrogen and phosphorus from wastewater, are all very specific for aiding microalgae growth and even small changes in these physical and chemical parameters cut the growth efficiency by more than 50% (Novoveská et al., 2023). To solve this, better photobioreactors (PBRs) with automatic controls for monitoring and adjustments fix this, like for example, the hybrid designs that mix wastewater nutrients automatically (Ting et al., 2017). The harvesting step can be challenging as both, biodiesel production and water remediation require a separation of microalgae from water, with centrifugation being energy intensive, flocculation clumping issue and hence an increase in chemical costs, along with high filtration clogging possibilities. This issue if not resolved, accounts for 20–50% of total costs. But, optimization with low-energy flocculants or magnets can increase recovery by more than 90%, and more cost efficiently (Hassanien et al., 2023; Ugwu et al., 2025). Large scale systems for real factories often struggle (Novoveská et al., 2023) with the designing like ponds or tanks, and high upfront costs (around more than $200 k/ha), with steady efficiency in changing waste. Running pilot plants to test these systems before fully scaling them as per use can help optimize and figuring out best possible solutions in designing (Piyatilleke et al., 2024). Genetically modifying microalgae DNA, altering its absorption capability for more pollutant consumptions like two times the heavy metals (Ranjbar and Malcata, 2022), and producing extra lipids (around 20–50%) for successful biodiesel amounts, directly improves overall process efficiency (Kumar et al., 2020). Simultaneously, this creates tough strains with fast growth rate potential. Therefore, the further research can be focussed on targeting genes for better remediation and fuel production (Hassanien et al., 2023). In addition to the conditions of cultivation, the composition variations in the wastewater imparts a big challenge for achieving high and constant microalgal biomass production. Photosynthetic activity, biomass productivity, and nutrient-removal activity can all be impacted by seasonal changes in temperature, solar radiation, rainfall, and flow of wastewater. The lower productivity is typically seen during colder seasons and extreme hot seasons. There have been cases where stress factors increased the biomass growth but if the stress is given very high and excess nutrient limitation occurs then it can affect productivity of biomass, lipid and FAME profiles reducing the yield of biodiesel (Ramírez Mérida and Rodríguez Padrón, 2023). Pathogenic microorganisms may raise biosafety issues when wastewater-grown biomass is meant for food, feed, agriculture, or other high-value applications, competing microorganisms such as bacteria, fungi, protozoa, and grazers can destabilise the culture but for biodiesel production many studies showed positive results in FAME profiles. Microalgal–bacterial consortia might also have the ability to improve nutrient removal and pathogen removal such as *Chlorella vulgaris*–bacterial systems have reported for showing enhanced removal of *Escherichia coli*, *Pseudomonas aeruginosa*, and *Enterococcus faecalis* under controlled aeration and cultivation conditions (Ruas et al., 2022). Furthermore, heavy metals such as cadmium, lead, chromium, nickel, copper, and zinc may inhibit photosynthesis and microalgal growth at elevated concentrations, so it beneficial to utilize microalgae that can remove these metals through adsorption, precipitation, and intracellular bioaccumulation. In a recent study it is seen that after microalgae is cultivated in wastewater, the pathogens and heavy metals were found to be at acceptable limit, only cadmium surpassed the limited threshold (Álvarez-González et al., 2023).

## 5 Transesterification process and optimization of the microalgal lipid

In transesterification the triglyceride molecules from microalgae are converted into biodiesel (Shanthini et al., 2025). From a stoichiometric standpoint, three moles of alcohol per mole of triglyceride is required. However, excess alcohol ratios are usually applied to facilitate complete conversion. The identification and relative distribution of FAMEs using GC–MS confirm the successful conversion of triglycerides into biodiesel (Tsikas, 2024). Figure 7 illustrates how the general chemical equation can be used to explain a simple transesterification process.

**Figure 7:**
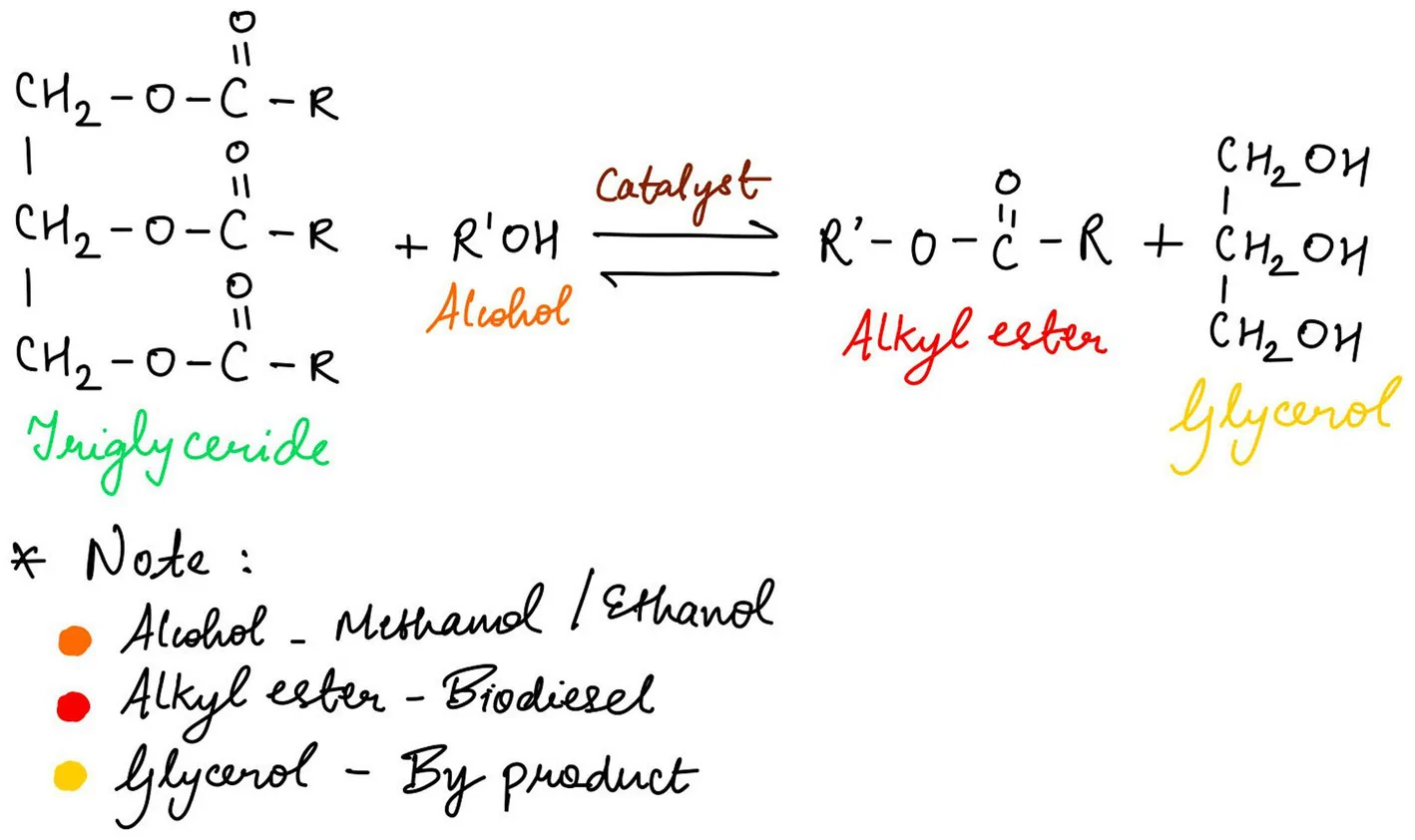
Basic equation for transesterification.

### 5.1 In-situ and ex-situ transesterification

In-situ transesterification, also called as direct transesterification, has gained importance as an energy efficient and cost-effective method where lipid extraction and transesterification occurs together in a single step (Oğuz et al., 2025). By eliminating separate oil extraction, it is gaining importance in sustainable biodiesel production. The main drawback is the water presence in the wet microalgal biomass which can lead to lower biodiesel yield. It can be carried out in the presence or absence of a catalyst under extreme conditions. Extra purification steps can be avoided for the end products in the absence of a catalyst (Pandey et al., 2024). It can be carried out using either one-step and two-step approaches. In the one-step approach, microalgal biomass is directly reacted with methanol and a single acid or alkali catalyst to produce FAMEs in a single reaction whereas in the two-step in-situ transesterification, it involves successive addition of acid and alkali catalysts which provided better control over conversion enhancing transesterification (Oğuz et al., 2025). A technoeconomic study demonstrated that direct transesterification as compared to a conventional two-step process showcased a significant reduction in biodiesel production cost from 18.2 $ kg^−1^ to 12.5 $ kg^−1^. Sensitivity analysis indicated biomass source, solvent recycling, biodiesel yield and plant capacity as critical cost-influencing factors. Under further optimization, the unit production cost was reduced to 3.5 $ kg^−1^ (Lee et al., 2019).

*Ex-Situ* Transesterification also called as indirect or conventional transesterification involves extraction of microalgal lipids using organic solvents and which are then converted to alkyl esters using homogeneous or heterogeneous catalysts. This process employs acid, base or even biocatalysts largely influenced by the fatty acid content and reaction conditions. Catalyst choice and operating conditions have a major impact on conversion efficiency. Adjusting and optimizing catalyst type, temperature, pH and reaction duration significantly improves catalytic activity and higher biodiesel yields (Goh et al., 2019). Lipid extraction generally involves three sequential steps: solvent penetration through the cell wall and membrane into the cytoplasm, dissolution of lipids to form lipid–solvent complexes and outward diffusion of these complexes out of the cells (Alam et al., 2019). The quantity of lipids extracted from microalgae relies on the effectiveness of cell disruption and solvent selection. Microalgal cell walls are made of multiple structural components that hinder solvent penetration. The presence of water reduces the formation of lipid-solvent complexes and needs to be removed to increase lipid yield (Nazloo et al., 2022). A comparative study by (Tran et al., 2012) was done using the two routes: ex-situ (M-I) and in-situ (M-II) transesterification for biodiesel production from *Chlorella vulgaris* ESP 31. The results showed that M-II achieved a higher biodiesel conversion of 97.3 wt% oil whereas M-I reached 72.1 wt% oil which indicates that the indirect route offers better control over extraction and reaction steps but the additional processing steps contribute to overall cost and energy demand.

## 6 Effective catalysts used for transesterification

Transesterification is dependent on the catalyst applied for biodiesel synthesis. Catalysts are essential to increase the speed of the reaction while minimizing unwanted side reactions. This makes it important to understand catalyst properties and have a detailed, careful comparison for selecting the most suitable one for a reliable, efficient and high yield biodiesel production. Catalysts help in overcoming immiscibility problems between oil and alcohol, improving surface contact and resulting in a stable reaction. Based on their nature, they are broadly grouped into chemical (homogeneous, heterogeneous) and biological (enzyme-based) systems (Mandari and Devarai, 2021).

### 6.1 Homogenous catalysts

The transesterification of triglycerides is often done using homogeneous catalysts. These catalysts are commonly used due to their high reaction efficiency and simplicity because they are in the same phase as the reactants due to which there is better molecular interaction resulting in rapid reaction kinetics. However, their limitations such as post reaction separation and purification steps need to be addressed. Both acids and alkalis are frequently used for transesterification. Base catalysts such as sodium hydroxide, sodium methoxide, and potassium hydroxide are commonly selected and generally preferred over acid catalysts as they operate effectively under moderate conditions, lowering energy input and making it more practical at a commercial scale (Vicente et al., 2003; Ghedini et al., 2021). (Cercado et al., 2018) explored biodiesel production by evaluating the three different alkali catalysts - KOH, NaOH and LiOH. NaOH and KOH achieved better FAME conversion than LiOH. Integrating microwave and ultrasound further improved the efficiency by about 5% and reduced reaction time by 50%. Another recent study by (Bhuana et al., 2020) utilized sulfuric acid as an acid catalyst for the microwave-assisted *in situ* transesterification of *Chlorella* sp. Under optimized conditions of 370 W microwave power and 0.2 M catalyst concentration. The process achieved about 63% yield confirming its suitability with the help of response surface methodologies such as Box–Behnken optimization. Research validates their effectiveness in biodiesel synthesis. Nevertheless, catalyst recovery and product purification remain technical and economic challenges (Mandari and Devarai, 2021).

### 6.2 Heterogeneous catalysts

Heterogeneous catalysts have gained significant attention in green biodiesel research as homogeneous catalysts face various disadvantages such as saponification due to sensitivity with water and fatty acids, toxic nature along with several others. Further development and continued research on parameters such as oil feedstock composition, catalyst structure and calcination conditions will strengthen their application in biodiesel synthesis. Process variables such as stirring rate, methanol ratio and temperature remain critical for higher performance (Oyekunle et al., 2023). These catalysts are prepared from natural or waste derived materials making them safer, reusable and less corrosive. They are capable of producing high FAME yields above 98% when derived from sources such as calcined biowastes. However, limitations such as catalyst leaching and deactivation need to be tackled effectively (Jayakumar et al., 2021; Welter et al., 2023). A recent study by (Rahman et al., 2019) explored mixed oxide catalysts for biodiesel production from *Nannochloropsis oculata*. These Bi_2_O_3_ impregnated ZrO_2_ catalysts prepared via sol–gel method achieved high FAME yield under optimized conditions although oxidative stability is a key concern that needs to be addressed. Another study involved the use of zirconium-doped HZSM-5 where hydrothermal liquefaction of *Scenedesmus obliquus* was done under nutrient stress where a decrease in protein content was observed along with enhanced biodiesel production and increased lipid content (Mustapha et al., 2022). Calcium Oxide (CaO) is a widely used heterogeneous catalyst for microalgal biodiesel production due to their high catalytic efficiency, low cost and ease of recovery. To promote greener production, researchers are emphasizing on producing CaO from discarded shell waste (Zhang et al., 2013). The application of CaO/hydrotalcite for in-situ transesterification of *Nannochloropsis* sp. was successfully applied where addition of n-hexane improved lipid recovery and high stirring speeds increased reaction performance reaching 38.36% biodiesel yield and low FFA levels (Mirzayanti et al., 2023). Waste-derived catalysts made from animal bones, eggshells, and oyster shells present a viable way to lower catalyst costs and increase the value of waste materials in the manufacturing of biodiesel. While animal bones mostly include calcium phosphate and hydroxyapatite, whose catalytic characteristics rely on their composition and preparation circumstances, oyster shells and eggshells are calcium-rich sources that can be calcined into CaO. These heterogeneous catalysts have potential benefits over traditional homogeneous catalysts in terms of catalytic recovery, reusability, and decreased wastewater production during catalyst separation. However, catalyst loading, calcination temperature, surface basicity, oil properties, methanol-to-oil ratio, and reaction temperature all affect their catalytic activity and biodiesel production. Currently, eggshell-derived CaO seems to offer the best overall balance of catalytic activity, low catalyst loading, availability, and reusability among the three forms of catalysts. Under ideal circumstances, eggshell-derived CaO has generated biodiesel yields of roughly 94–97%, often with catalyst loadings of 1–3 weight percent. A comparative investigation found that a homogeneous base catalyst produced a 98% biodiesel yield, whereas calcined eggshell-derived CaO produced a 97% yield at 1.5 weight percent catalyst loading. While the eggshell-derived catalyst provided easier recovery and lessened the need for aqueous neutralisation and washing, the homogeneous catalyst produced a little higher yield and required a shorter reaction time, but the yield difference was negligible (Ayodeji et al., 2018; Tavizón-Pozos et al., 2025).

#### 6.2.1 Nano catalysts

Nanocatalysts fall under the category of heterogeneous catalysts where it offers improved surface properties resulting in enhanced reactivity and better catalytic performance (Shaik and Kumari, 2025). They are available in different forms such as mesoporous, magnetic, carbon-based and metal oxide structures. Among these, metal oxides and carbon-based materials are increasingly used due to their stability and adaptability with different microalgal feedstocks (Mofijur et al., 2020). Different techniques such as gas condensation, impregnation, chemical vapour deposition and microwave-assisted techniques are commonly used to prepare nano catalysts with desired properties (Ambat et al., 2018). Nanocatalysts have been successfully applied in microalgal biodiesel offering enhanced properties and reusability. For example, in a study by (Abazari et al., 2024), 32.7% lipid content was obtained from *Scenedesmus* sp. under optimized culture conditions, and later followed by the use of a magnetic NiFe_2_O_4_@SiO_2_/MgO nanocatalyst achieving a high biodiesel yield of 95.3%. Similarly, a CaO nano-biocatalyst showed efficient biodiesel conversion resulting in a 86.5% FAME yield from *Monoraphidium* sp. NCIM 5585, which under optimized growth conditions produced a high lipid content of 47.3% (Pandey et al., 2025).

### 6.3 Enzymatic catalysts for bio-transesterification

Enzymatic catalysts, also called as lipase-based catalysts form the second category of the types of catalysts used in transesterification. Lipases, also referred to as triacylglycerol hydrolases assist in the degradation of triglycerides and are further categorized into two types which are free lipase and immobilized lipase (Xia et al., 2023). Enzymatic-transesterification provides high-quality biodiesel by improving selectivity and avoiding saponification. Yet, their large-scale deployment is limited due to their high cost and slower reaction rate (Fattah et al., 2020; Farouk et al., 2024) In biodiesel production, lipase enzymes accelerate the transesterification process by following a ping-pong bi-bi kinetic pathway ensuring efficient conversion into esters. However, it is important to understand the tolerance to methanol and water content which is critical for catalytic activity (Norjannah et al., 2016). Lipase immobilization methods are generally divided into two major types: physical methods which includes adsorption and entrapment, and chemical methods involving covalent attachment and cross linking (Mota et al., 2022). Numerous studies highlight the potential of enzymatic transesterification of microalgal biomass. In one such study by (López et al., 2015), biodiesel production from *Nannochloropsis gaditana* using intracellular Rhizopus oryzae lipase, where increasing lipid purity and solvent selection by the use of tert-butanol instead of n-hexane enhanced biodiesel conversion to nearly 80%. In another experimental study, (Guldhe et al., 2015) found that immobilized *Pseudomonas fluorescens* lipase helped in achieving high biodiesel yields from *Scenedesmus obliquus* of upto 91% with stepwise methanol addition. Table 5 provides a comparison of the key technologies applied across cultivation, harvesting, lipid extraction and transesterification based on their reported performance, economic and energy considerations and applicability to biomass grown in wastewater.

**Table 5:** Summary of various technologies used in different stages of microalgal biodiesel production, including their reported performance, advantages and limitations based on their stage.

Stage	Technology	Reported performance	Advantage/limitation	Reference
Cultivation	Open raceway pond	Low volumetric productivity; $0.1–0.3/m^2^ CAPEX	Low cost; handles large wastewater flows; poor controllability	Chisti (2007)
Cultivation	Closed PBR	2–5 × higher biomass (10–20 g/m^2^/day)	Precise control; low contamination risk; high CAPEX ($50-200/m^2^)	Chisti (2007); Skifa et al. (2024)
Cultivation	Mixotrophic (wastewater carbon)	54 mg/L/day lipid productivity, *Chlorella vulgaris*	Relies on residual organic carbon present in the treated effluent	Liang et al. (2009)
Cultivation	Two-phase N-starvation	Lipid >60%; productivity 117 → 204 mg/L/day, *Nannochloropsis* sp.	Biomass and lipid formation occur separately: involves an additional stage	Rodolfi et al. (2008)
Harvesting	Centrifugation	17–96% recovery	Rapid; applicable to most strains; energy-intensive (0.8–20 kWh/m^3^)	Barros et al. (2015)
Harvesting	Chemical flocculation	80–99% recovery	Economical and energy-saving; potential biomass contamination	Vandamme et al. (2013)
Harvesting	Bioflocculation (sludge-assisted)	100% harvesting in 8.5 h	Chemical costs avoided; applicable to wastewater	Tiwari and Prajapati (2025)
Harvesting	Magnetic nanoparticle separation	90–95% recovery	Rapid; nanoparticle cost and recyclability are concerns	Markeb et al. (2019)
Extraction	Bligh and Dyer / Folch (solvent)	Up to 612 mg/g lipid, *Chlorella vulgaris*	High recovery; toxic chlorinated solvents	De Bhowmick et al. (2024)
Extraction	Mechanical (bead-mill/microwave)	Most effective of 5 pretreatments tested	Little to no solvent required; energy intensive for large scale operation	Lee et al. (2010)
Extraction	Supercritical CO₂	Selective for neutral lipids	No solvent residue; high-pressure systems increase CAPEX	Halim et al. (2012); Askari et al. (2022)
Transesterification	Homogeneous base/acid catalyst	Up to 98% FAME conversion	Base: fast, FFA-sensitive; acid: slower, FFA tolerant	Mandari and Devarai (2021)
Transesterification	Heterogeneous (CaO/nano)	86.5–95.3% biodiesel yield	Reusable, inexpensive precursors; risk of leaching and deactivation	Abazari et al. (2024); Pandey et al. (2025)
Transesterification	In-situ (direct)	97.3 wt% oil conversion; cost $18.2 → $12.5/kg	Fewer unit steps, cost effective; water sensitive	Tran et al. (2012); Lee et al. (2019)

## 7 Advantages and uses of microalgae across industrial applications along with techno-economical analysis (TEA) and life cycle assessment (LCA)

Rising CO_2_ emissions from industrial and human activities have become a major concern due to its serious environmental and health impacts. In response to this, the role of microalgae is becoming a major research focus as its cultivation acts as a green alternative for absorbing CO_2_ and converting it into valuable products like biomass and biofuels. Integrating microalgal cultivation with wastewater treatment increases the viability of microalgae for carbon capture too as resource efficiency is enhanced along with lesser input requirements. Unlike traditional crops, microalgae do not depend on fertile soil or seasonal growth patterns. Their adaptability to grow in saline water and various environmental conditions underscores their importance in long term carbon mitigation strategies (Rajkumar et al., 2025). When microalgae is cultivated in systems such as open ponds or closed photobioreactors, carbon fixation rates are higher than those of conventional crops due to the focus on novel strategies, such as using industrial flue gases for carbon utilization, enhancing its practical relevance (Singh et al., 2025; Cheng et al., 2025). A recent review shows that strains such as *Chlorella vulgaris* and *chlorella* sp. displayed biomass outputs up to 1.21 g/L/d and CO_2_ fixation rates. Among the strains analysed, *Nannochloropsis oceanica* achieved highest biodiesel productivity of 0.36 g/L/d with strong cost efficiency of $1.5/L and favourable energy returns of around 13.5 (Rashad et al., 2025). Different microalgal species showcase specialised biochemical and metabolic properties that make them suitable for the production of diverse renewable fuels. *Nannochloropsis oceanica* contains about 12.1% saponifiable lipids in its dry biomass which can be efficiently converted into biodiesel through catalytic esterification with up to 85% biodiesel purity, highlighting its adaptability for bioenergy applications (Gong et al., 2020). Hydrogen is a further renewable fuel option in addition to biodiesel, with *C. reinhardtii* capable of photobiological hydrogen generation. As hydrogenase-related limitations reduce production efficiency, modifications targeting photosystem II have been implemented (King et al., 2022). Microalgae assist in removing nitrogen and phosphorus through photosynthetic growth, reduction of BOD, CO₂ fixation, and oxygen release. Their cell surfaces and extracellular polymers promote biosorption and biodegradation of xenobiotic compounds, while heavy metals can be immobilised by algal biomass. Their growth in nutrient-rich domestic, industrial and agricultural wastewaters lowers chemical inputs and enables simultaneous resource recovery and treatment (Kundu et al., 2024).

Techno-economic analyses are carefully evaluated to move microalgal biodiesel from laboratory research to industrial application. One study explores whether biodiesel derived from microalgae can be realistically implemented at an industrial scale. Cultivation of *Phaeodactylum tricornutum* in 80,000 m^3^ photobioreactors across 15.247 ha, produced 1,811 tonnes of biomass and 171,705 L of biodiesel annually. The production costs were estimated at €2.01 kg^−1^ for biomass and €0.33 L^−1^ for biodiesel, with an ROI of 10% and a 10-year payback period, proving potential for medium-term commercial deployment (Branco-Vieira et al., 2020). Microalgal biomass is increasingly utilised for the production of omega-3 lipids, pigments, proteins, and bioactive peptides targeting nutraceutical and functional food sectors but large-scale supply remains constrained by cultivation system choice, contamination risk as well as downstream processing costs. Closed photobioreactors are often required for premium products due to which there is an increase in capital and operational expenditure (Barkia et al., 2019). Large-scale studies show that microalgae-based biodiesel can be technically feasible but high costs limit its wider use. For a 500-ha production facility, capital costs range from US$ 128–245 million, while annual operating costs are US$ 7.7–16.4 million, depending on location. Despite these high investments, microalgae diesel can reduce CO₂ emissions by nearly 65–70% compared to fossil diesel microalgae systems which proves they offer significant sustainability advantages although further cost reduction is necessary for large-scale commercial adoption (Roles et al., 2021). A study highlights that ANN-assisted optimization offers a reliable pathway for improving biodiesel yield and process sustainability. Under experimentally optimised conditions, using 50 g/L *Chlorella vulgaris* biomass and sulfuric acid catalysis resulted in 97.46% FAME yield. ANN predictions further enhanced performance, producing 98.12% yield, demonstrating that ANN integration enhances efficiency while reducing reliance on extensive experimental iterations (Snaina et al., 2025).

Life cycle assessment (LCA) is an important tool for evaluating the environmental sustainability of microalgae-based biodiesel by considering energy consumption, greenhouse gas (GHG) emissions, resource requirements, and other environmental impacts throughout the production chain. For microalgal biodiesel, the system boundary generally includes cultivation, harvesting, dewatering and drying, lipid extraction, transesterification, biodiesel purification, and downstream processing. LCA studies indicate that the environmental performance of microalgal biodiesel is strongly dependent on the energy and material inputs associated with these individual stages rather than on biodiesel conversion alone. Therefore, identifying the major energy- and emission-intensive operations is essential for improving the overall sustainability of the process (Chamkalani et al., 2020). Cultivation is a significant contributor to energy consumption and GHG emissions in microalgal biodiesel production, heavily influenced by methods such as mixing, pumping, and artificial lighting. The choice between open ponds and closed photobioreactors affects environmental profiles because even while closed systems may require more resources, they offer better control and less contamination (Guccione et al., 2014). Using wastewater can lessen environmental impacts compared to freshwater, although electricity demand remains high. Harvesting, dewatering, and drying stages are crucial environmental hotspots, necessitating energy-intensive processes like centrifugation and thermal drying. Improvements in biomass concentration and low-energy harvesting are vital for reducing the environmental footprint. Lipid extraction and transesterification also contribute significantly to environmental burden via energy and chemical inputs. The GHG emissions during the entire lifecycle depend on energy sources and inputs. LCA studies emphasize the necessity of integrated approaches to minimize energy demands and environmental impacts, advocating for the use of renewable resources and efficient processing for enhanced sustainability (Chamkalani et al., 2020; Liu et al., 2025). LCA of biodiesel produced from *Chlorella vulgaris* using a hybrid cultivation system combining a photobioreactor and an open raceway pond. In a production scale of 1 ton of biodiesel, the integrated process included microalgal pretreatment, process of transesterification, post-treatment, combustion of microalgal biodiesel, and recycling of generated waste achieved up to a 42% reduction in CO₂ emissions compared with conventional petroleum diesel production and combustion (Kim et al., 2022). Table 6 presents the reported economic, energy and environmental impacts across the microalgal biodiesel systems reviewed above.

**Table 6:** Reported production costs, energy inputs and environmental impacts of microalgal biodiesel systems.

Parameter	System/route	Reported value	Reference
Cultivation capex	Closed PBR	$50–200/m^2^	Skifa et al. (2024)
Production cost	General	$4–10/kg biomass; $2–6/L biodiesel (vs. $0.8/L fossil diesel)	Maroušek et al. (2023)
Transesterification cost	In-situ vs. conventional (optimised)	$18.2 to $12.5/kg (optimised: $3.5/kg)	Lee et al. (2019)
Facility-scale economics	500-ha facility	Capex $128 - 245 M; opex $7.7–16.4 M/yr.; 65–70% lower CO₂ vs. fossil diesel	Roles et al. (2021)
Pilot-scale economics	80,000 m^3^ PBR, *Phaeodactylum tricornutum*	€2.01/kg biomass; €0.33/L biodiesel; 10% ROI	Branco-Vieira et al. (2020)
Cultivation energy demand	Open pond vs. closed PBR	1–2 MJ/kg biomass (ORP) vs. 5–10 MJ/kg (PBR)	Herrera et al. (2020)
GHG reduction	Hybrid PBR–ORP, *Chlorella vulgaris*	42% lower CO₂ vs. conventional diesel	Kim et al. (2022)
Harvesting/extraction cost share	General	20–50% of total production cost	Hassanien et al. (2023); Ugwu et al. (2025)

## 8 Integration of artificial intelligence (AI) and machine learning (ML) for optimizing wastewater to biodiesel systems for microalgae

When we talk about enhancing or improving microalgae wastewater to biodiesel systems, the important implementation cannot be said complete without Artificial Intelligence (AI) and Machine Learning (ML) integration (Mafat et al., 2024; Sable and Ikar, 2024; Nyoni et al., 2024). They improve microalgae wastewater treatment process and biodiesel production via some specific domains like predictive control, real-time adjustments, along with better algae strain design, which can increase outputs by 20 to 40% when compared to manual techniques (Abuhasheesh et al., 2025). Long Short-Term Memory (LSTM) (Le et al., 2019) and similar neural networks analyse general sensor data like pH (6.5–9), temperature (25–35 °C) dissolved oxygen (DO) around 5–15 mg/L, are collectively used to predict growth rates (Wang and Zhang, 2024) like 0.3–0.6 per day as well as lipid levels (usually 30–55% dry weight) and this automatically adjusts LED lighting and CO₂ levels for optimal results (Yang et al., 2025). ML models for deep learning are used to simulate photobioreactor conditions and this is to maximize nutrient removal (Carrasquero-Ferrer et al., 2025) of around 90–98% N/P and achieve biodiesel potential, by processing camera images to detect contamination ahead of the point of ruins as well as for fine-tuning aeration (Li Z. et al., 2025) for biomass growth (1.5–2.5 g/L-day) in various wastewater kinds. Decision-trees and other alike AI optimization tools improve and enhance productivity compared to traditional controls (Lim et al., 2022). Optimal harvesting techniques like flocculation or centrifugation (Rahman et al., 2024) are studied by reinforcement learning (RL) software which work like smart coach that undergo multiple combinations of harvesting and then getting “rewards” for successful results, and this is responsible for reducing energy while maintaining high levels of recovery rates (Sulaiman and Al-Raweshidy, 2025) along with suitable lipids and fat profiles for biodiesel from variable wastewater. RL and some ML also approach optimization by analyzing factors, for example, algae size (5–50 μm) or flocculant dose (10–50 mg/L), and centrifuge force (3000-8000 g) (Wu et al., 2025). Meanwhile generative AI (Kanwal et al., 2025) for designing strain aligns with analyses of biological data and picking out, suggesting gene editing targets, and creating wastewater-tolerant strains with improved lipid production. This shared learning across research sites speeds up the development for cost-effective biodiesel pathways (Li P. et al., 2025). With sensor-based monitoring and real-time process management, photobioreactors can effectively incorporate AI and ML models. Predictive models like LSTM, artificial neural networks, and random forest algorithms use data from sensors that measure pH, temperature, dissolved oxygen, light intensity, CO_2_ concentration, and biomass as input variables. While optimisation algorithms can suggest changes to CO_2_ supply, illumination, mixing, and fertiliser feeding, these models can forecast biomass growth, nutrient removal, and lipid buildup. Statistical measures like R^2^ (Coefficient of Determination), RMSE (Root Mean Square Error), and MAE (Mean Absolute Error) should be used to assess the model’s performance, and independent experimental cultivation data should be used to validate it. However, there are still significant obstacles to adopting AI-based real-time optimisation in large-scale microalgal cultivation systems, including differences in wastewater composition, sensor reliability, data availability, and limited pilot- and industrial-scale validation. Technologies that have reached industrial deployment differ significantly from those still under laboratory or pilot testing, especially in terms of immediate commercial applicability. Technologies such as open raceway ponds, closed photobioreactors, centrifugal and flocculation-based harvesting, solvent extraction of lipids, and homogeneous or heterogeneous catalytic transesterification, including CaO produced from waste shells have reached commercial sale application and are supported by established by economic and performance data (Chisti, 2007). Several technologies examined here are still at an early stage of development, with most evidence limited to laboratory and or small pilot-scale applications. Approaches such as CRISPR/Cas9-mediated strain engineering and RNA interference for improving lipid accumulation have primarily been tested in contained photobioreactor systems, but their ecological safety remains tied to engineered containment mechanisms such as genetic kill switches rather than established regulatory frameworks under field conditions (Saha et al., 2025; Čelešnik et al., 2016). Similarly magnetic nanocatalysts and bio-derived nano-CaO catalysts indicate high FAME conversion under laboratory optimization, but their scalability to long-term industrial productions is not yet established (Abazari et al., 2024; Pandey et al., 2025). The application of AI and ML is increasingly extending beyond prediction toward active process optimization, including LSTM -based forecasting for biomass growth, reinforcement-learning frameworks for improving harvesting methods and generative design of improved strains. Although encouraging results have been reported, they remain largely at the proof of concept or pilot stage, with comparatively little demonstration under variable and constant changing conditions of full-scale wastewater-integrated plants (Abuhasheesh et al., 2025; Wu et al., 2025; Kanwal et al., 2025). Establishing comparable performance at industrial scale remains a key hurdle for commercial implementation.

## 9 Conclusion

With analysis of the various cultivation techniques and exploring different biofuel production routes, catalytic transesterification as well as the themes of waste water treatment systems, the review sheds light to one of the rising industrial applications with respect to microalgae integration. By incorporating AI- driven solutions for optimization of parameters along with the advanced characterization tools, it’s become an easy and cost-effective solution toward sustainability with scalability. Several shells like oyster shells and other calcium carbonate rich bones and shells can prove to be of sustainable introduction in form of heterogenous catalysts in transesterification, serving the purpose of an eco-friendlier approach as CaO-rich sources that is formed after calcination at 700–900 °C. Various scientific advancements in water remediation and biofuel production systems using microalgae are explored in depth and in turn, aligning us with the future scopes of improving research through the integration of genetically improved strains, AI monitored operations and large-scale adaptability of the processes and efficiency. The scope of improvement lies in environmentally benefitted systems with multi-use factories for combined operations like harvesting, biodiesel production, biogas and protein by product utilization, all while incorporating natural CaCO_3_ sources for CaO production as discussed above. Despite recent advances, important gaps remain, particularly the lack of standardized methods for strain purity, consistency in C: N nutrient ratios and establishing uniform specifications for biodiesel quality (Magyar et al., 2023; Akram et al., 2025). Considerable variation in the scope and assumptions of techno-economic analysis hinders meaningful comparison across studies. Long-term performance data from pilot and industrial systems remains limited, particularly for wastewater-based cultivation using real (non-synthetic) effluents under fluctuating environmental conditions. Likewise, repeated-cycle studies on heterogeneous catalysts, particularly their reusability and susceptibility to leaching, also requires greater attention. Research priorities should include methodological consistency through standardized reporting, validate performance over longer outdoor operating periods using real wastewater, and assess economic and environmental performance together across all major processing steps within a unified system boundary. Future research should focus on converting innovations in the lab into standard, economically feasible pilot and industrial systems. Enhancing strains using genetic and adaptive methods to boost biomass and lipid production is a top objective, as is systematically standardising wastewater composition and pre-treatment to increase repeatability across culture systems. It is also necessary to develop low energy harvesting and lipid extraction techniques to lower production costs and downstream energy consumption. To improve resource utilisation and financial feasibility, more work is required in the biorefinery setting on cultivation, wastewater treatment, biomass separation, lipid extraction, biodiesel synthesis, and co-product recovery. The primary objectives of ongoing development of reliable, reusable, and scalable heterogeneous catalysts, particularly waste-derived catalysts, should be to reduce leaching, enhance recyclability, and maintain catalytic activity throughout repeated operation. Simultaneously, machine learning and artificial intelligence-based optimisation should transition from laboratory prediction to real-time process management and adaptive optimisation under changing effluent and operating conditions. Lastly, pilot-scale validation under realistic and evolving wastewater conditions accompanied by techno-economic and life-cycle assessments should be used to assess the process’s dependability, energy efficiency, environmental performance, and commercial viability. When combined, these goals offer a way to move microalgal biodiesel research from the lab to integrated, environmentally friendly industrial biorefineries.
